# The Effects of Cardiopulmonary Fitness on Executive Functioning or Academic Performance in Students from Early Childhood to Adolescence? A Systematic Review

**DOI:** 10.3390/jfmk10030254

**Published:** 2025-07-04

**Authors:** Markel Rico-González, Ricardo Martín-Moya, Francisco Javier Giles-Girela, Luca Paolo Ardigò, Francisco Tomás González-Fernández

**Affiliations:** 1 Department of Didactics of Musical, Plastic and Corporal Expression, University of the Basque Country, UPV-EHU, 48940 Leioa, Spain; markel.rico@ehu.eus; 2 Department of Physical Education and Sports, Faculty of Education and Sport Sciences, Campus of Melilla, University of Granada, 52006 Melilla, Spain; javiggr@hotmail.com (F.J.G.-G.); ftgonzalez@ugr.es (F.T.G.-F.); 3 Department of Teacher Education, NLA University College, 0166 Oslo, Norway

**Keywords:** education, physical activity, academic performance, executive functioning, VO_2_max

## Abstract

**Background:** Cardiovascular fitness has been proposed as a key factor influencing executive functioning and academic performance during childhood and adolescence. However, the extent and consistency of this relationship remain unclear across diverse populations and educational contexts. This systematic review aimed to evaluate whether cardiovascular fitness, particularly measured through VO_2_max, is consistently associated with improvements in executive function and academic performance among students from early childhood to adolescence. **Methods:** A systematic search of PubMed, Web of Science, SPORTDiscus, and ProQuest Central was conducted up to 15 November 2022. Studies were included if they examined correlations between VO_2_max and cognitive or academic outcomes in students from preschool to high school. Methodological quality was assessed using the MINORS checklist. **Results:** Out of 271 identified studies, 12 met all inclusion criteria. Evidence suggests that higher VO_2_max is generally associated with improved executive function domains such as attention, working memory, and inhibitory control, as well as academic performance indicators including mathematics and reading scores. Neurophysiological studies also indicate links between cardiovascular fitness and brain structure/function. However, the strength and specificity of these associations vary across studies due to methodological differences, limited sample diversity, and inconsistent control for confounders. **Conclusions:** Cardiovascular fitness appears to have a positive, albeit complex, relationship with cognitive function and academic performance in youth. Future research should adopt longitudinal and experimental designs to clarify causal pathways and consider moderating factors such as sex, age, and psychosocial variables.

## 1. Introduction

Education, from childhood through adolescence, is one of the most important challenges for humanity. In fact, its importance is reflected in its inclusion as one of the 17 Sustainable Development Goals outlined by the United Nations in Agenda 2030 [1]. Recognising this significance, researchers are continuously exploring methodologies and associated factors that improve children’s academic performance [2]. Academic achievement is generally characterised by students’ attitudes toward school and their capacity to attain positive educational outcomes [3].

From a general viewpoint, one of the paradigms related to academic performance is the cardiovascular fitness hypothesis [4,5]. This term is commonly defined as the capacity to generate energy during intense physical activity [6]. The impact of occasional physical exercise on academic success has garnered increasing scientific interest, resulting in a growing body of research. Several studies indicate that children with higher levels of physical fitness tend to perform better academically compared to their less fit peers [5,7]. Moreover, higher cardiorespiratory fitness has been associated with enhanced performance in various cognitive tasks [8], even when the physical activity involves simple movement [9].

While VO_2_max is a widely used measure of cardiovascular fitness, its direct relationship with cognitive performance and academic achievement appears to be limited [10]. Several studies suggest that the benefits of physical activity on cognition are primarily mediated through neurocognitive mechanisms rather than VO_2_max per se [11]. For instance, overall physical activity and fitness levels have been associated with improvements in executive functions [12], which are crucial for learning and academic success [5,7,13]. Such enhancements are believed to result from exercise-induced neuroplasticity, including increased hippocampal volume, enhanced synaptic plasticity, and elevated levels of brain-derived neurotrophic factor (BDNF) [14]. Furthermore, emerging evidence suggests that moderate-to-vigorous physical activity, regardless of its impact on VO_2_max, can lead to short-term boosts in cognitive processes related to attention and information processing speed [15]. Specifically, aerobic exercise interventions focusing on complex movement patterns and coordination have been shown to produce structural brain changes in regions involved in learning and memory, such as the hippocampus and prefrontal cortex although individual genes may influence this association [16]. These neurocognitive benefits may underpin the observed correlations between physical activity and academic performance, highlighting the importance of considering mechanisms beyond cardiorespiratory capacity alone.

Research has increasingly focused on understanding the relationship between academic achievement and executive functions, with numerous studies exploring how cognitive processes influence school performance. In this regard, evidence suggests that physical fitness, particularly aerobic capacity, plays a significant role in supporting key cognitive functions such as attention, concentration, memory, and information processing speed [7,12,17,18] such as concentration. These findings underscore the importance of cardiovascular health in educational contexts, spanning from childhood through adolescence, as it can directly impact learning abilities and academic success [12].

Despite the accumulating evidence pointing toward positive outcomes, the research landscape remains complex, with results often showing variability and inconsistency across different studies. Some investigations report strong correlations, while others find minimal or no effects, contributing to ongoing scientific debate about the causative mechanisms and the strength of these associations [19]. This heterogeneity highlights the need for critical appraisal of the existing literature to prevent the adoption of ineffective or unsupported educational practices [5].

Therefore, the primary goal of this systematic review is to evaluate whether cardiovascular fitness, particularly measured through VO_2_max, is consistently associated with improvements in executive function and academic performance among students from early childhood to adolescence. This review aims to serve as a valuable resource for educators and policymakers by providing reliable, evidence-based insights. Such guidance is crucial for designing interventions that genuinely enhance student learning outcomes and avoid relying on unverified protocols or misconceptions that could hinder progress or lead to wasted resources.

## 2. Materials and Methods

### 2.1. Experimental Approach to the Problem

A systematic review was performed in accordance with PRISMA (Preferred Reporting Items for Systematic Reviews and Meta-Analyses) guidelines [20] and the guidelines for performing systematic reviews in sports sciences [21].

### 2.2. Information Sources

A systematic search of four databases (PubMed, Web of Science, SPORTDiscus, and ProQuest Central) was performed to identify articles published prior to 21 November 2022.

### 2.3. Search Strategy

The PICO (Patient, Problem, or Population—Intervention or Exposure—Comparison, Control, or Comparator—Outcome[s]) design was used to provide an explicit statement of the question [20]. Where possible, the search was limited to scientific articles/journals and language (see exclusion criteria number 5). The author was not blinded to journal names or manuscript authors. The following search terms were used (see Table 1):

(child* OR school-age OR student*) AND (correlation* OR associate* OR relation*) AND (“cardiopulmonary fitness” OR VO_2_max OR “oxygen consumption”) AND (school OR “elementary education” OR “elementary school” OR “primary education” OR “primary school” OR “secondary education” OR “high school”)

### 2.4. Eligibility Criteria

To systematically identify relevant information from the articles, the authors first extracted key information from each article, including titles, authorship, publication dates, and the databases from which they were retrieved. This information was organised into an Excel spreadsheet (Microsoft Corporation, Redmond, WA, USA), facilitating efficient data management. To ensure data integrity, duplicate entries were carefully identified and removed. The remaining articles were screened against predefined inclusion and exclusion criteria, as summarised in Table 1. This process ensured that only studies pertinent to our research questions were selected.

Additionally, relevant articles that were not initially identified through the search process were re-evaluated using the same criteria. Any new studies meeting the inclusion standards were then incorporated into the review and marked as “included from external sources” for transparency. To promote transparency and maintain rigorous standards throughout our review, we followed the PRISMA 2020 guidelines, which offer a comprehensive framework for conducting systematic reviews. This process involved carefully defining and justifying our inclusion and exclusion criteria to ensure a consistent and unbiased selection of relevant studies.

### 2.5. Data Extraction

Data extraction was prepared using an Excel spreadsheet in accordance with the Cochrane Consumers and Communication Review Group’s data extraction template. The spreadsheet was used to assess inclusion and exclusion requirements for all selected studies. Full-text articles that were excluded from the analysis were recorded with reasons for exclusion. All records were stored in the spreadsheet.

### 2.6. Data Items

After identifying all published articles, the outcome domains considered the most important for interpreting the review’s conclusions and providing a rationale for the labelling were all variables that provide any information about intervention: sample, aim, assessment, statistical analysis, other independent variables, correlation or prediction, and highlights.

### 2.7. Quality of Studies

The methodological quality was assessed using the methodological index for non-randomised studies (MINORS) [22]. The MINORS scale is a list that contains 8 essential points and it is expanded to 12 points when the studies are to be treated are comparative. In this case, it was assessed considering 9 items (out of 18 points) due to the non-applicability (NA) of 3 of them. The score that each section receives can be from 0 to 2, depending on the quality obtained for each point.

## 3. Results

### 3.1. Identification and Selection of Studies

**Figure 1:**
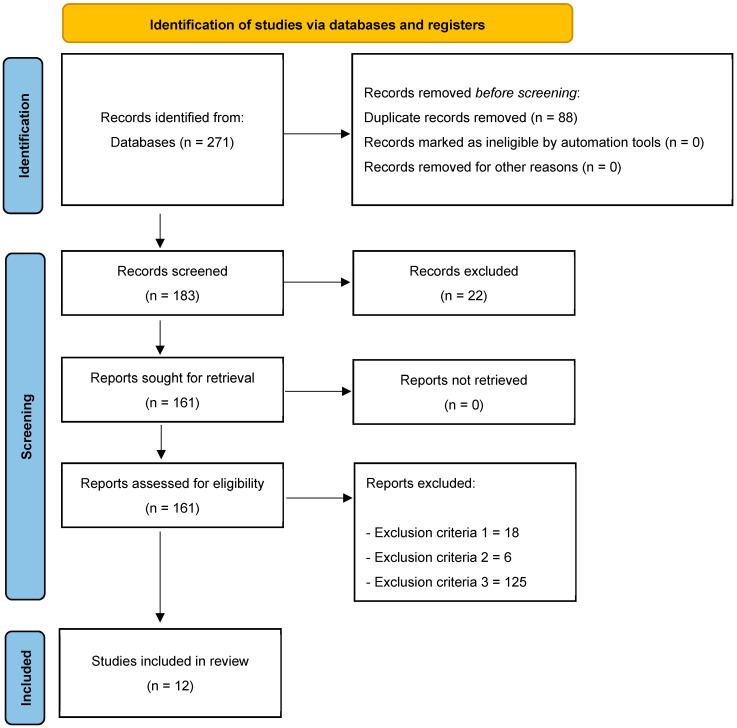
Flow diagram of the study.

A total of 271 (Web of Science: 139; PubMed: 14; ProQuest: 59; SPORTDiscus: 59) original articles were found, of which 88 were duplicates. Thus, a total of 183 unique articles were identified. After checking titles and abstracts, 22 articles were excluded because they did not meet the inclusion criterion number five. The full text of the remaining 161 articles was then analysed; 18, 6, and 125 articles were excluded because they did not meet exclusion criteria one, two, three, and four, respectively. Thus, 12 articles met all the inclusion criteria and were included in the final qualitative synthesis (Figure 1).

### 3.2. Quality Assessment

Using the MINORS checklist, the quality assessment is performed in Table 2. The items are scored 0 (not reported), 1 (reported but inadequate), or 2 (reported and inadequate). Red = poor quality, yellow = moderate quality, and green = good quality, with the global ideal score being 16 for non-comparative studies and 24 for comparative studies.

### 3.3. Study Characteristics

The characteristics of studies were extracted and clustered into Table 3 and Table 4 (for statistics).

The 12 studies included in this systematic review (Table 3, Table 4 and Table 5) collectively demonstrate nuanced relationships between cardio-pulmonary fitness (VO_2_max) and cognitive/academic outcomes in youths aged 6–24 years. VO_2_max was significantly associated with diverse outcomes beyond executive function and academic performance, including physical self-concept (PSC) [18,26], health-related quality of life (HRQOL) [23,25], mental health [27], and inhibitory control [8]. Notably, negative correlations emerged between VO_2_max and depression/body image concerns [26], suggesting broader psychosocial implications of fitness. A higher VO_2_max correlated with improved concentration [7,17,18], working memory [7,12], and academic achievement (e.g., arithmetic, reading) [4,5]. Neurophysiological mechanisms were implicated, such as reduced theta oscillation power [8] and increased cortical thickness in the frontal regions [4]. Some studies reported no direct VO_2_max–academic performance links (e.g., verbal working memory in [12], highlighting domain-specific effects). Boys consistently exhibited higher VO_2_max and physical fitness levels than girls [23,27], yet girls showed superior concentration and HRQOL [23]. VO_2_max correlated more strongly with concentration in children aged 9–10 years [23] and with cognitive flexibility in adolescents [17]. Studies used diverse tools (e.g., Posner task for attention [17], Paced Auditory Serial Addition Test for working memory [7]), limiting cross-study comparability. While most used indirect measures (e.g., Léger test), direct calorimetry [4,17] yielded more precise but less feasible data. Sex, BMI, socioeconomic status, and lifestyle factors (e.g., screen time [26]) frequently influenced outcomes but were inconsistently controlled. Effect sizes were typically small to moderate (e.g., β = 0.16–0.21 for concentration [23]; η^2^ = 0.06–0.15 for physical self-concept [16]). Despite statistical significance (*p* < 0.05), clinical/educational relevance remains tentative, as most variance in academic outcomes was unexplained (e.g., r^2^ = 0.043 in males [5]).

## 4. Discussion

This systematic review critically assessed whether cardiovascular fitness, particularly measured via VO_2_max, is consistently associated with executive function and academic performance in children and adolescents. While a positive association emerges in several studies, the evidence is far from uniform. Our contribution lies in reporting observed effects and offering a comparative, contextual and methodological analysis that reveals important nuances and limitations within the literature.

Among the 12 studies reviewed, associations between cardiovascular fitness and cognitive function appear domain-specific. Notably, visuospatial working memory, attention control, and information processing showed the most consistent links to VO_2_max. Meijer et al. [12] reported such effects with the largest sample (*n* = 814), while verbal working memory and interference control showed no relationship. This pattern suggests that the impact of aerobic fitness may be selective, benefiting those executive functions more closely tied to sensorimotor coordination and attentional resources.

Another important finding is the role of motor task complexity. Ryu et al. [5] and Canepa et al. [7] proved that complex motor tasks involving coordination and sequencing predicted academic performance more reliably than simple aerobic tasks. This suggests that cognitive engagement during movement may play a critical mediating role. Rather than aerobic fitness per se, the cognitive demands embedded in the movement patterns may stimulate neurocognitive adaptation. This hypothesis also aligns with findings from neurophysiological studies such as Hsieh et al. [8], who reported that VO_2_max levels predicted lower modulation in midfrontal theta oscillations, a neural marker of executive control.

A noteworthy gender-related pattern emerged from several studies. In Ryu et al. [5], fitness measures explained significantly more variance in academic performance for girls than for boys. While these findings require cautious interpretation due to possible sampling or cultural effects, they suggest a potential sex-based differential sensitivity to the cognitive benefits of fitness. Whether physiological, motivational, or neurodevelopmental factors drive these differences remains an open question.

Despite some convergence in results, methodological differences severely limit the comparability of studies. VO_2_max was assessed using at least five protocols, ranging from indirect field tests to direct calorimetry. Cognitive outcomes were equally diverse, from reaction-time paradigms to academic grades. This heterogeneity highlights the lack of standardisation in the field and complicates any attempt to aggregate results meaningfully. More concerning is the inconsistent control for confounding variables such as socioeconomic status, pubertal stage, dietary habits, and screen time. These factors influence cognitive and physical development, yet were often neglected in statistical models.

Beyond cognition, several studies pointed to broader psychosocial benefits of cardiovascular fitness, including improved self-esteem, mood regulation, and health-related quality of life (HRQOL) [23,25,26]. While these variables were not always directly tied to academic outcomes, they likely operate as indirect facilitators of learning, suggesting that future research should consider multi-layered models incorporating physical, cognitive, emotional, and contextual dimensions.

Our synthesis also reveals that studies often lack a theoretical framework to integrate biological, psychological, and behavioural mechanisms. Without such a framework, the field risks producing isolated findings that resist generalisation. For example, while thinner cortical regions in high-fit children have been associated with better math performance [4], the developmental and causal implications remain speculative.

### Limitations of the Study and New Lines of Research

This review is limited by the relatively small number of eligible studies (*n* = 12) and the considerable variability in study design, measurement tools, and analytical rigour. Most studies were cross-sectional, restricting causal inference. Only a minority included neurophysiological measures or intervention components. Additionally, while the inclusion criteria were carefully applied, the scope of the review may have excluded relevant studies due to language or indexing limitations.

Future research should adopt longitudinal or experimental designs with standardised VO_2_max assessments and well-validated cognitive tasks. Studies that integrate neurocognitive, psychosocial, and educational outcomes within unified models should be given priority. More attention is also needed to test the mediating role of motor task complexity and gender-specific trajectories. Finally, it is essential to control for confounding factors such as nutrition, screen time, and sleep, which may otherwise obscure the real effects of fitness on cognition and learning.

## 5. Conclusions

Cardiovascular fitness supports cognitive and academic functioning in youth, particularly in tasks requiring attention, processing speed, and visuospatial working memory. However, these benefits are not universal and should not be overstated. Rather than aerobic fitness alone, the cognitively demanding nature of certain physical activities may drive improvements in executive function. Gender differences, task type, and contextual factors further modulate these associations.

In light of these findings, educators, and policymakers should be cautious in interpreting the fitness–academic link. Promoting physical activity remains essential, but interventions to enhance academic performance should consider incorporating structured, complex motor tasks that challenge the body and the mind. The next generation of research must go beyond correlational evidence and toward an integrated understanding of how movement, cognition, and development intersect in real-world educational settings.

## Figures and Tables

**Table 1:** Inclusion/exclusion criteria of included studies.

Item	Inclusion	Exclusion	Search Coherence	Justification
Population	Students from preschool to high school.	Children out of preschool or compulsory education.	Child school-age, student*	Health habits from the first years of life are crucial for a healthy lifestyle.
Intervention or Exposure	VO_2_max was measured in students during education settings.	VO_2_max was not measured. VO_2_max was measured outside of an educational setting (e.g., activities outside of school hours).	school, “elementary education”, “elementary school”, “primary education”, “primary school”, “secondary education”, “high school”	Education settings are suitable environments because a lot of children go to these settings every day.
Outcome[s]	Relationship between VO_2_max and cognitive factors, executive functioning, well-being, mood, emotions, self-concept, and self-esteem.	No VO_2_max was measured. VO_2_max was measured but was related to health (e.g., BMI, fat mass, back pain, spinal posture, blood pressure), physical fitness performance, lifestyle (e.g., sedentary time, time watching TV, and mobile, tablets), and anthropometrical parameter, or illness (e.g., anaemia, diabetes).	“Cardiopulmonary fitness”, VO_2_max, “oxygen consumption”	Because the scientific literature has tried to correlate cognitive variables and VO_2_max.
Study design	Correlation study or, at least, correlations were included.	Correlations were not assessed.	correlation*, associate*, relation*	
Other criteria	Peer reviewed, original, full-text studies written in English, Italian, or Spanish.	Written in another language or non-peer-reviewed original full-text studies.	-	Articles evaluated through peer-review process, writing in English, as the main common language, and authors’ mother tongue.

**Table 2:** Quality assessment of selected articles with the MINORS checklist.

		Köble et al. [23]	Zurita-Ortega et al. [24]	Gálvez Casas et al. [25]	Wengaard et al. [17]	Meijer et al. [12]	Mayorga-Vega et al. [18]	Hsieh et al. [8]	Canepa et al. [7]	Delgado-Floody et al. [26]	Liang et al. [27]	Ryu et al. [5]	Chaddock-Heyman et al. [4]
1	A clearly stated aim	2	2	2	2	2	2	2	2	2	2	2	2
2	Inclusion of consecutive patients	2	2	2	2	2	2	2	2	2	2	2	2
3	Prospective collection of data	2	2	2	2	2	2	2	2	2	2	2	2
4	Endpoints appropriate to the aim of the study	2	2	2	2	2	2	2	2	2	2	2	2
5	Unbiased assessment of the study endpoint	0	0	0	0	0	0	0	0	0	0	0	0
6	Follow-up period appropriate to the aim of the study	2	2	2	2	2	2	2	2	2	2	2	2
7	Loss to follow-up less than 5%	0	0	0		2		2		0			0
8	Prospective calculation of the study size	2	2	2	2	2	2	2	2	2	2	2	2
Additional criteria in the case of comparative study													
9	An adequate control group						2						
10	Contemporary groups						2						
11	Baseline equivalence of groups						2						
12	Adequate statistical analysis	2	2	2	2	2	2	2	2	2	2	2	2
	Total MINORS score	14	14	14	14	16	20	16	14	14	14	14	14
	Maximum possible Score	18	18	18	16	18	22	18	16	18	16	16	18

**Table 3:** Correlations between oxygen consumption and other factors.

Reference	Sample	Aim	Assessment	Statistical Analysis	Other Variables	Correlations or Predictions	Highlights
Köble et al. [23]	Nº children: 6533 Schools: 15 Country: Germany (Mean age n/a) (Range 6 ÷ 10 years) Level: n/a Grade: n/a	Examining the physical fitness (PF) levels of primary school children of different ages and different genders, and determining the associations among PF, concentration, and HRQOL	Assessment of mass with scale and height with stadiometer. PF assessment (push-ups, curl-ups, standing long jump, handgrip, 20-m multistage shuttle run, and maximal oxygen consumption [VO_2_max] estimation with Progressive Aerobic Cardiovascular Endurance Run). Concentration assessment withd2-R test HRQOL assessment with German KINDL questionnaire.	Independent *t*-test to assess differences over age, gender, and mass, and univariate analysis of variance to assess differences over PF, concentration, and HRQOL. Pearson correlation analysis and multiple linear regression analysis to assess association among PF, concentration, and HRQOL.	n/a	PF increases with age in all dimensions but VO_2_max in girls. Boys show higher PF than girls in all dimensions but curl-ups at age ≥ 7 years. Concentration increases at age 7 ÷ 9 years. Girls show higher concentration and HRQOL than boys. Higher VO_2_max is associated with higher concentration and HRQOL at age 9–10 years.	Cardiopulmonary fitness is important for improved concentration and better HRQOL in 6 ÷ 10 year-age children.
Gálvez Casas et al. [25]	Nº children: 298 Schools: n/a Country: Spain (Mean age 9.76 ± 1.36 years) (Range 8 ÷ 12 years) Level: n/a Grade: n/a	Analyse the relationship between aerobic capacity and quality of life.	The aerobic capacity (VO_2_max) was assessed through Course-Navette test. The quality of life was assessed through KIDSCREEN-10 Index.	Differences in the variables studied based on sex were studied using a simple variance analysis (one-way ANOVA). To study the quality of life as a function of the CA level (low, medium and high), a simple analysis of variance (one-way ANOVA) was performed, where the aerobic capacity level was introduced as a fixed factor and the quality of life as the dependent variable.	Sex	Levels of the quality of life were significantly greater in children with higher level of VO_2_max in comparison with those with lower level (*p* = 0.001). Boys with a high level of aerobic capacity showed higher levels of quality of life in relation to their peers with a low level (*p* < 0.001). Girls showed significant differences were detected between those who had a high level of aerobic capacity and their peers with a low level (*p* = 0.031).	The results of this study show that school children with a higher level of aerobic capacity have a higher level of quality of life.
Zurita-Ortega et al. [24]	Nº children: 515 Schools: 27 Country: Chile (Mean age 10.5± 0.5 years) (Range 10–11 years) Level: primary Grade: 4–5	To analyse the relationship between physical conditions, body mass index (BMI), level of physical activity, and self-esteem.	VO_2_max through Course-Navette test. Hand pressure through dynamometry. Vertical jump through maximal covered distance BMI through weighing machine. PA level through PA questionnaire.	Structural equation model.	n/a	A negative relationship between BMI and maximal oxygen consumption, jumping ability, physical activity, and self-esteem. Finally, self-esteem was positively related to physical activity engagement.	Self-esteem was related to physical activity variables.
Wengaard et al. [17]	Nº children: 54 Schools: 2 Country: Norway (Mean age 17.9 ± 0.9 years) (Range n/a) Level: n/a Grade: n/a	Investigating the association of physical fitness, measured as VO_2_max, muscle mass, weekly training, and cognitive function in the executive domains of selective attention in healthy male high-school students.	Body composition (total mass and muscle per cent mass) assessment with body composition analyser. Direct VO_2_max custom-protocol assessment with indirect calorimetry. Selective attention assessment with Posner cue paradigm-based visual cognitive test (reaction time [RT] after no cue, valid cue, or invalid cue presentations). Relevant background information (PA frequency, daily video game playing time, and self-perceived alertness level) assessment with questionnaire.	Linear mixed model analysis to assess association between (PA frequency, daily video game playing time, and self-perceived alertness level adjusted) VO_2_max and visual cognitive test performance. Linear mixed model analysis to assess association between muscle per cent mass and PA frequency and visual cognitive test performance.	Heart rate (HR) ADHD diagnosis, dyslexia and daily nicotine use.	Higher VO_2_max is associated with shorter RT after valid cue or invalid cue. No association between muscle per cent mass and PA frequency and visual cognitive test performance.	Cardiorespiratory fitness is associated with cognitive performance in healthy male high-school students in the executive domains of selective attention.
Meijer et al. [12]	Nº children: 814 Schools: 22 Country: Netherlands (Mean age 9.16 ± 0.65 years) (Range 7.44 ÷ 11.14 years) Level: Grade: 3–4	Investigating the relationship between cardiovascular fitness and executive functioning in a large sample of healthy children.	VO_2_max estimation with Léger test information processing, attention processes, and interference control assessment with adapted Attention Network Test Verbal working memory (WM) assessment with Digit Span Task. Visuospatial WM assessment with Grid Task. Motor inhibition assessment with Stop Signal Task. Intelligence quotient (Information and Block Design) assessment with Wechsler Intelligence Scale for Children III.	Mixed regression analysis to assess effect of VO_2_max on neurocognitive components.	Sex, age, socioeconomic status (SES), and participation in sports.	Higher VO_2_max is associated with higher information processing and control, visuospatial WM, and attention efficiency. There is no association between VO_2_max and verbal WM, attention accuracy, and interference control.	There is a relationship between cardiovascular fitness and a specific set of executive functions and lower-level neurocognitive functions. Cardiovascular fitness is important for the overall health of school-aged children.
Mayorga-Vega et al. [18]	Nº children: 75 Schools: 1 Country: Spain (Mean age 11.1 ± 0.4 years) (Range n/a) Level: n/a Grade: 6	Investigating the effect of an eight-week PF training on the physical self-concept (PSC) in primary education children in a physical education setting.	PSC assessment with Spanish Physical Self-Description Questionnaire. PF assessment with EUROFIT test battery.	Multivariate analysis of covariance to assess training effect on PSC and PF. Univariate analysis of covariance + Bonferroni to assess interactions.	Body mass. Height.	Training improves PF. In experimental group, PSC does not change. In control group, PSC worsens.	The improvements in PF are not accompanied by major changes in PSC, even though the training maintains the experimental group’s previous PSC, which worsens in the control.
Hsieh et al. [8]	Nº children: 171 Schools: n/a Country: USA (Mean age 8.9 ± 0.6 years) (Range 8–9 years) Level: n/a Grade: n/a	Investigating the association between cardiorespiratory fitness and midfrontal theta oscillations evoked by a flanker task in children, and seeking whether midfrontal theta oscillation mediates the relationship between cardiorespiratory fitness and inhibitory control task performance.	Direct VO_2_max modified Balke-protocol assessment with indirect calorimetry. Inhibitory control assessment with modified Eriksen flanker task with lower and higher cognitive demand. Electroencephalographic activity recording.	Pearson correlation to assess association between VO_2_max and inhibitory control score. Pearson correlation to assess association between VO_2_max and modulation of theta (4–7 Hz) oscillatory power.	Pubertal timing SES Intelligence quotient Assessment for presence of eventual physical exercise-exacerbated health issues.	Higher VO_2_max correlates with higher inhibitory control score. Higher VO_2_max correlates with lower modulation of theta (4–7 Hz) oscillatory power.	Higher cardiorespiratory fitness is associated with better and more stable performance on a task that modulates inhibitory control. Higher cardiorespiratory fitness is associated with better top-down control and cortical communication, as reflected by midfrontal theta.
Canepa et al. [7]	Nº children: 40 Schools: n/a Country: Italy (Mean age 19.18 ± 6.18 years) (Range 10 ÷ 24 years) Level: n/a Grade: n/a	Investigating the correlation between the 12 min-walk/run test (12m-WRT) and the performance of students of distinct school-grade levels at two different WM-related tasks.	WM assessment with education years-normalized PASAT and SDMT. VO_2_max estimation with 12m-WRT.	Independent *t*-test to assess VO_2_max differences over gender. Correlation to assess association between VO_2_max and WM over school grade (primary, secondary, and university).	HR. Rate of perceived exertion.	Males show higher VO_2_max than females. Higher VO_2_max correlates with higher WM scores in all participants pooled. Higher VO_2_max correlates with higher PASAT scores in primary and secondary school students. Higher VO_2_max correlates with higher SDMT scores in university students.	12m-WRT is associated with WM performance, showing different correlations with PASAT and SDMT according to the school-grade level. This might be due to the different effects that aerobic fitness has on specific neural substrates during development and opens avenues to research new tools able to monitor the health of the brain in young subjects.
Caamaño-Navarrete et al. [28]	Nº children: 617 Schools: n/a Country: Chile (Mean age 12 years) (Range 10 ÷ 14 years) Level: n/a Grade: n/a	Investigating the association of PSC with physical status (PF and mass) psychological well-being (depression and body image) and lifestyle (PA, nutritional level and screen time [ST]) in schoolchildren.	PSC assessment with questionnaire Depression assessment with Child Depression Questionnaire. Body image assessment with Body Shape Questionnaire Physical activity (PA) levels assessed with PA Questionnaire for children. Lifestyle (Mediterranean diet adherence, ST, and extra-school PA time) assessment with Krece Plus test. PF (VO_2_max estimation with Léger test, handgrip muscle strength with hand dynamometer) assessment. Assessment of mass with scale, height with stadiometer, and waist circumference with tape.	Spearman correlation to assess association among PSC, lifestyle, PF and anthropometry. Linear regression to assess association between PSC and any single variable.	n/a	Lower PSC correlates with higher depression, lower body image, lower extra-school PA time, higher ST, and lower VO_2_max. Higher PSC correlates with higher Mediterranean diet adherence. Lower PSC and PF correlate with lower body image.	Promoting healthy lifestyles among children should be a target of community- and school-based interventions to promote PSC.
Liang et al. [27]	Nº children: 253 Schools: 3 Country: China (Mean age n/a) (Range years 12–13) Level: n/a Grade: n/a	Investigating the association among PF, diet, and mental health.	Body height and mass assessment with tester. PF assessment with Chinese National Student Physical Fitness Standard (cardiorespiratory fitness test, vital capacity with spirometer, running 800 m for girls and 1000 m for boys, standing long jump, 50-m run, and sitting forward flexion test with tester). Mental health assessment with Chinese Middle-School Student Mental Health Scale. Energy intake and dietary calcium intake assessment with weighed 7-day food diary.	T-test and analysis of variance to assess differences over gender and body mass index (BMI). Pearson correlation and stepwise multiple regression to investigate relationships among PF, mental health, and dietary intake.	n/a	Boys are taller and heavier, with higher BMI and PF than girls. Girls are more flexible than boys. Low-BMI adolescents show higher PF, energy intake, and dietary calcium intake than normal-BMI. Higher cardiorespiratory fitness and calcium intake correlate with higher mental health. Cardiorespiratory fitness and calcium intake explain 8.4% of mental health changes.	Adequate calcium intake and the improvement of cardiorespiratory fitness in adolescents aged 12–13 are essential for the good development of their mental health.
Ryu et al. [5]	Nº children: 110 Schools: 1 Country: South Korea (Mean age 9.0 ± 0.3 [females], 8.9 ± 0.3 [males] years) (Range years n/a) Level: Grade: 4	Investigating associations between anthropometrics, PA, simple, and complex fitness tests and academic achievement.	Body mass index and fat (%) assessment with stadiometer and skinfolds. Korean literacy, math, social study, science, and English assessment. Daily steps count with pedometer. Direct VO_2_max Bruce-protocol assessment with indirect calorimetry. Sit-up (repetitions #), sit and reach (cm), standing long jump (cm), 50-m run (s), and hand grip (kg with dynamometer) test performance assessments. Illinois agility (s), soda pop hand (s), and foot (s) test performance assessments.	T-test to assess differences between females and males. Pearson correlation to assess associations between anthropometrics, PA, fitness tests, and academic achievement scores. Multi-predictor regression models for summed academic achievement scores.	n/a	Higher Illinois Agility, soda pop, hand and foot test scores correlate with higher achievement scores in females. Higher simple fitness tests correlate a little with higher academic achievement.	Regression modelling of body composition, PA, and fitness tests account for 30.5% of the variation of summed academic achievement scores in females but only 4.3% in males.
Chaddock-Heyman et al. [4]	Nº children: 48 Schools: n/a Country: USA (Mean age 9.96 ± 0.64 [lower fit], 9.98 ± 0.61 [higher fit] years) (Range years n/a) Level: n/a Grade: n/a	Investigating mutual inter-relationships between VO_2_max, brain cortical thickness, and academic performance.	Direct VO_2_max maximal-protocol assessment with indirect calorimetry. Magnetic resonance imaging (MRI) based brain cortical thickness assessment. Reading, spelling, and arithmetic assessment with wide range achievement test (WRAT-3).	Multivariate analysis of variance to examine association between aerobic fitness and brain cortical thickness. Secondary univariate analysis of variance to examine difference in cortical thickness over different aerobic fitness levels. Independent *t*-test to compare WRAT-3 score over different aerobic fitness levels. Pearson correlation to assess associations between cortical thickness and academic achievement.	Intelligence quotient. Presence of attentional disorders (Attention-Deficit Hyperactivity Disorder). Pubertal timing SES.	Higher aerobic fitness correlates with lower grey matter thickness. Lower grey matter thickness correlates with higher arithmetic performance.	Aerobic fitness may play an important role in cortical grey matter thinning in youth and, consequently, improve arithmetic performance.

Note: n/a = not applicable. BMI = body mass index; HRQOL = health-related quality of life; PA = physical activity; PASAT = Paced Auditory Serial Addition Test; PF = physical fitness; SDMT = Symbol Digit Modalities Test; VO_2_max = the maximum rate of oxygen consumption.

**Table 4:** Tests used, significance levels, and effect size.

Reference	Test Used	Significance Level	Effect Size
Köble et al. [23]	ANOVA, multiple linear regression	*p* < 0.001	β = 0.16 (concentration) β = 0.21 (HRQOL) Adjusted r^2^ = 0.078 − 0.106 r^2^ = 0.078 − 0.106
Zurita-Ortega et al. [24]	Student’s *t*-test for independent samples, Pearson’s correlation coefficient	*p* < 0.05	N/A
Gálvez Casas et al. [25]	One-way ANOVA with Bonferroni post hoc comparisons	*p* = 0.001 (overall), *p* < 0.001 (boys), *p* = 0.031 (girls)	N/A
Wengaard et al. [17]	Linear mixed model	*p* = 0.011 (invalid cue), *p* = 0.048 (valid cue)	N/A
Meijer et al. [12]	Mixed regression analysis	*p* < 0.001 (information processing and control, IPC), *p* = 0.001 (visuospatial working memory, VWM), *p* = 0.039 (attention efficiency, AE)	d = 0.14 (IPC), d = 0.12 (VWM), d = 0.08 (AE)
Mayorga-Vega et al. [18]	MANCOVA, ANCOVA with Bonferroni adjustment	*p* = 0.05 (overall), *p* = 0.003 (physical appearance), *p* = 0.02 (strength), *p* = 0.03 (self-esteem), *p* = 0.003 (sit-ups), *p* = 0.05 (20-m shuttle run)	η^2^ = 0.15 (physical appearance), η^2^ = 0.10 (strength), η^2^ = 0.08 (self-esteem), η^2^ = 0.14 (sit-ups), η^2^ = 0.06 (20-m shuttle run)
Hsieh et al. [8]	Pearson correlation, Hierarchical linear regression	*p* = 0.034 (congruent accuracy), *p* = 0.025 (incongruent accuracy), *p* = 0.049 (standard deviation of reaction time, SDRT), *p* = 0.052 (coefficient of variation of reaction time, CVRT), *p* < 0.001 (theta power)	β = 0.16 (congruent accuracy), β = 0.17 (incongruent accuracy), β = −0.14 (SDRT), β = −0.15 (CVRT), β = −0.31 (theta power)
Canepa et al. [7]	Independent *t*-test, Pearson correlation, Partial correlation, One-way ANOVA	*p* = 0.004 (VO_2_max–Paced Auditory Serial Addition Test [PASAT]), *p* = 0.003 (VO_2_max–Symbol Digit Modalities Test [SDMT]), *p* = 0.006 (SDMT in university students), *p* = 0.003 (PASAT in primary students), *p* = 0.040 (PASAT in secondary students)	N/A
Delgado-Floody et al. [26]	Spearman correlation, linear regression, Odds ratios (logistic regression)	*p* < 0.001 (VO_2_max and physical self-concept [PSC]), *p* = 0.01 (cardiorespiratory fitness [CRF] and PSC), *p* = 0.017 (Depression and PSC), *p* = 0.015 (PA and PSC)	N/A
Liang et al. [27]	T-test, ANOVA, Pearson correlation, stepwise multiple regression	*p* < 0.05 (cardiorespiratory fitness and mental health), *p* < 0.01 (calcium intake and mental health)	r^2^ = 0.084
Ryu et al. [5]	Pearson correlation, Multi-predictor regression modelling	*p*-values range from 0.003 to 0.045 for significant correlations (e.g., soda pop hand test with English: *p* = 0.003; Illinois agility with social studies: *p* = 0.012)	r^2^ = 0.305 (females), r^2^ = 0.043 (males)
Chaddock et al. [4]	MANOVA, ANOVA, Independent *t*-test, Pearson correlation	*p* = 0.017 (MANOVA), *p* = 0.034 (superior frontal), *p* = 0.025 (superior temporal), *p* = 0.021 (lateral occipital), *p* = 0.05 (math achievement), *p* = 0.04 (correlation with anterior and superior frontal thickness)	d = 0.62 (superior frontal), d = 0.65 (superior temporal), d = 0.69 (lateral occipital); other effect sizes not reported

**Table 5:** Strengths and weaknesses of each of the studies.

Reference	Strengths	Weaknesses
Köble et al. [23]	- Large sample size (6533 children) - Covered a diverse age range (6–10 years) - Found associations between VO_2_max and concentration and HRQOL	- Did not fully control for all potential confounders (e.g., socioeconomic status, nutrition)
Zurita-Ortega et al. [24]	- Found significant correlations between VO_2_max and quality of life - Highlighted gender differences in outcomes	- Smaller sample size (298 children), limiting generalizability
Gálvez Casas et al. [25]	- Linked VO_2_max with self-esteem and physical activity levels - Provided insights into holistic development	- Moderate sample size (515 children) - Focused on a narrow age group (10–11 years)
Wengaard et al. [17]	- Focused on older adolescents (average age 17.9), providing insight into a less-studied age group - Found associations between VO_2_max and selective attention	- All-male sample, limiting generalizability to females - Small sample size (54 participants)
Meijer et al. [12]	- Large sample size (814 children) - Assessed a broad range of cognitive functions - Found positive associations with information processing, visuospatial working memory, and attention efficiency	- Did not find associations with all cognitive functions (e.g., verbal working memory, interference control), indicating inconsistent effects
Mayorga-Vega et al. [18]	- Examined the impact of a physical fitness intervention, providing experimental evidence - Found improvements in physical fitness	- Small sample size (75 children) - Short intervention period (8 weeks) - No significant effect on physical self-concept
Hsieh et al. [8]	- Found associations between VO_2_max and inhibitory control - Included neurological correlates (midfrontal theta oscillations)	- Moderate sample size (171 children)
Canepa et al. [7]	- Covered a wide age range (10–24 years) - Found associations between VO_2_max and working memory	- Small sample size (40 students) - Wide age range may introduce confounding variables
Delgado-Floody et al. [26]	- Linked VO_2_max with mental health outcomes (e.g., depression, body image) - Large sample size (617 children)	- Focused on a specific age group (10–14 years), limiting broader applicability
Liang et al. [27]	- Found associations between cardiorespiratory fitness, calcium intake, and mental health - Provided culturally specific insights	- Moderate sample size (253 adolescents) - Cultural specificity may limit generalizability
Ryu et al. [5]	- Found that fitness tests accounted for a significant portion of academic achievement variance, especially in females	- Small sample size (110 children) - Gender differences noted but not fully explained
Chaddock et al. [4]	- Found neurological correlates (grey matter thickness) associated with aerobic fitness and arithmetic performance - Provided a potential neurological basis for cognitive benefits	- Small sample size (48 children) - Narrow age range (average age 9.96–9.98 years)

## Data Availability

Not applicable.
